# Supplementary material for: Advanced rechargeable aluminium ion battery with a high-quality natural graphite cathode
Source: Nat Commun. 2017 Feb 13;8:14283. doi: 10.1038/ncomms14283 (PMC5316828; doi:10.1038/ncomms14283)
Supplement: Supplementary Information — Supplementary Figures, Supplementary Tables, Supplementary Methods and Supplementary References [file ncomms14283-s1.pdf]

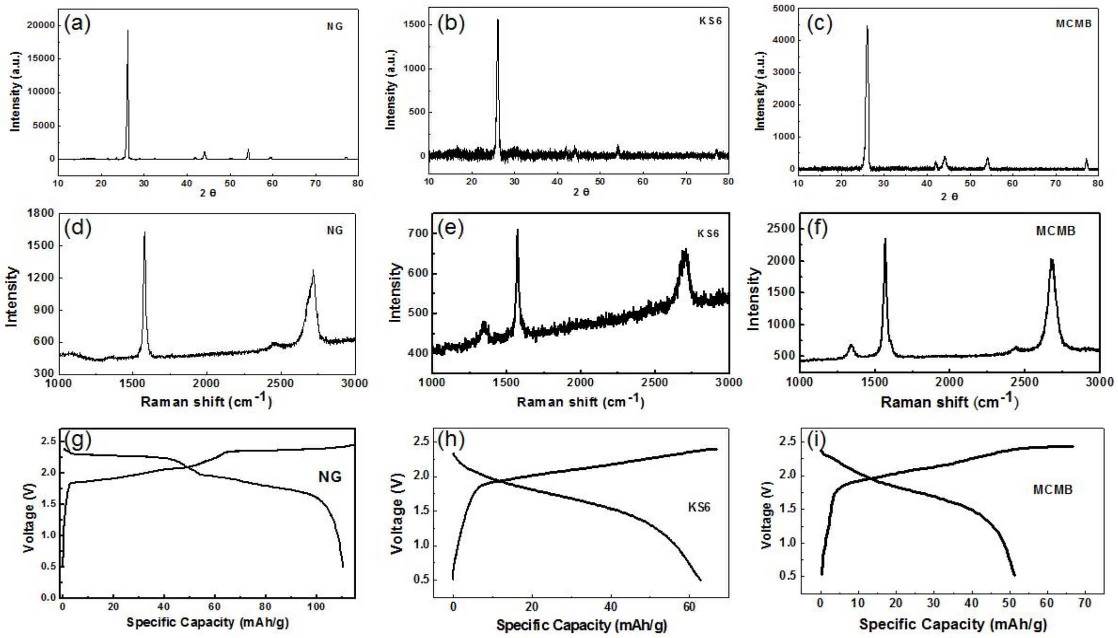

Supplementary Figure 1 | The electrochemical performances of different graphite materials related to their structure characterizations. The XRD spectra (a-c) and Raman spectra (d-e) of different kinds of commercial graphite including NG, KS6 and MCMB, respectively. (g-i) The Galvanostatic charge and discharge curves of Al ion battery using SP-1 graphite, KS6 and MCMB materials as cathode electrode charging and discharging at a current densities of 66 mA/g. All capacity of the batteries were recorded between charging and discharging voltage of 0.5 V and 2.45 V. The loading amount of NG, KS6 and MCMB are~ 4 mg cm^{-2}, only calculating weight of carbon materials without binder.

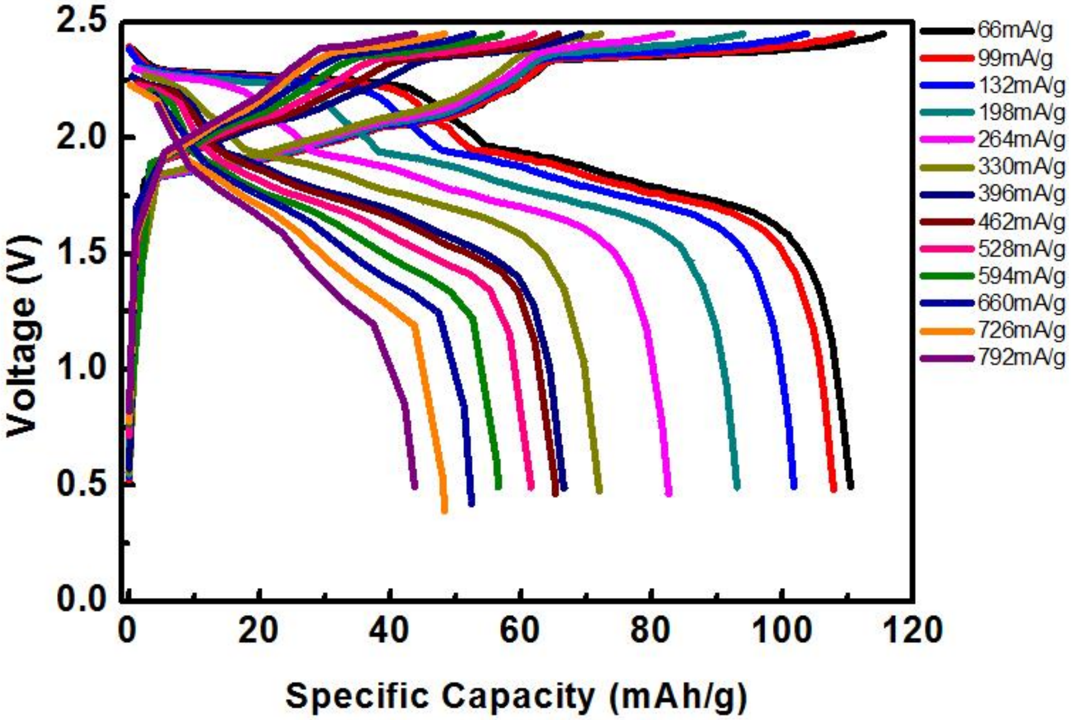

Supplementary Figure 2 | The corresponding galvanostatic charge and discharge curves of an Al/NG cell (see Figure 2 (b) in the main text). The loading amount of NG was 4 mg cm^{-2}, based on calculated weight of graphite without binder.

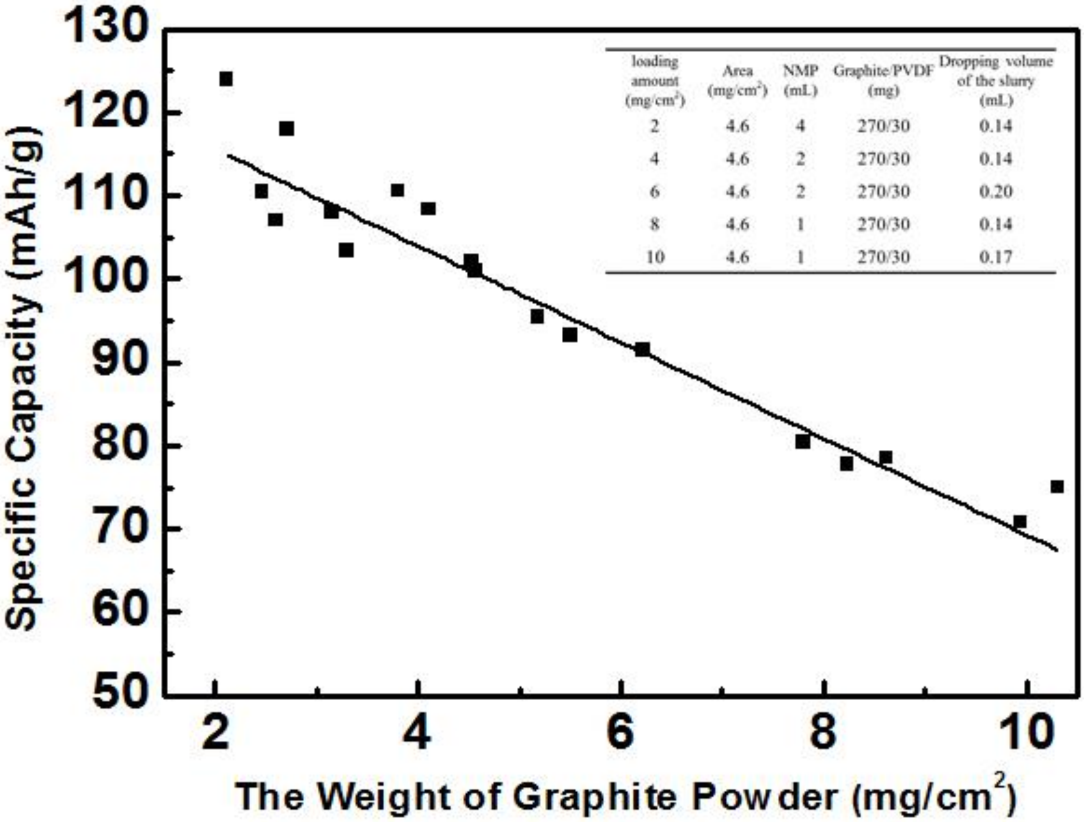

loading amount (mg/cm^2)	Area (mg/cm^2)	NMP (mL)	Graphite/PVDF (mg)	Dropping volume of the slurry (mL)
2	4.6	4	270/30	0.14
4	4.6	2	270/30	0.14
6	4.6	2	270/30	0.20
8	4.6	1	270/30	0.14
10	4.6	1	270/30	0.17

Supplementary Figure 3 | Al/NG cell capacity with different loading amounts of nature graphitic flake. The electrode area of all cell is 2 cm × 2.3 cm. First, two concentration of NG slurry was needed, which was controlled by addition of two volume of the NMP solvent (1 mL and 2 mL) into a constant amount of nature graphite (270 mg) and PVDF binder (30 mg). Then, the low loading amount (2 mg cm^{-2}), normal loading amount (4 to 6 mg cm^{-2}) and high loading amount (8 to 10 mg cm^{-2}) of electrodes was controlled by volume of slurry with 4 mL, 2 mL and 1 mL NMP solvent on the Cu foil, respectively. The table in the inset lists in detail the preparation quantities.

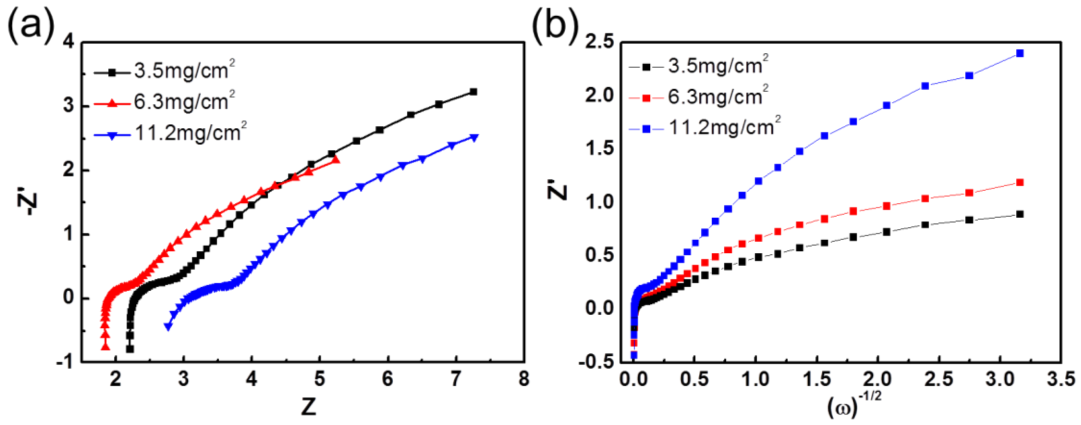

Supplementary Figure 4 | The EIS measurement of the batteries with different graphite loading amount. (a) EIS plots of the batteries with various graphite loading. (b) The relationship between Z' and $\omega^{-1/2}$ of the related EIS plots. The EIS measurement of the batteries was operated under charging at 2.4 V.

Diffusion can create an impedance, known as the Warburg impedance, which is related to the frequency (ω) of the potential perturbation. Equation 1 is the equation for the "infinite" Warburg impedance. With mobile anions, diffusion flux implies a Warburg-like impedance; this impedance is a $\omega^{-1/2}$ function.

$$Z = \sigma(\omega)^{-\frac{1}{2}}(1 - j) \quad (1)$$

On a Nyquist plot the infinite Warburg impedance appears as a diagonal line with a slope of 0.5. On a Bode plot, the Warburg impedance exhibits a phase shift of 45°. In the above equation, σ is the Warburg coefficient defined as:

$$\sigma = \frac{RT}{n^2F^2A\sqrt{2}C\sqrt{D}} \quad (2)$$

ω is radial frequency, D is diffusion coefficient of anions, A is surface area of the electrode, n is number of electrons transferred and C is bulk concentration of the diffusing species (moles cm^{-3}).

In Supplementary Figure 6 (b), we found that the slope of the relationship between Z' and $\omega^{-1/2}$ in the battery with high loading amount is higher than that with low loading amount. According to eq.2, this result indicates that the diffusion coefficient of anions in the battery with high loading is less than that with low loading. The result could be attributed to that the diffusion of chloroaluminate ions to graphite in depth might be reduced when the thickness of film is increased, resulting in reducing the specific capacity of the battery with higher graphite loading.

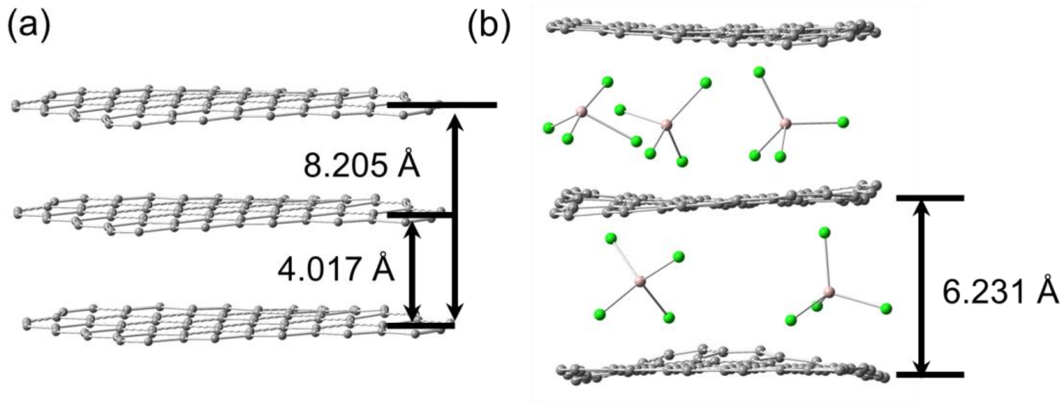

Supplementary Figure 5 | DFT models of the graphite with (a) three layers, and (b) intercalatants.

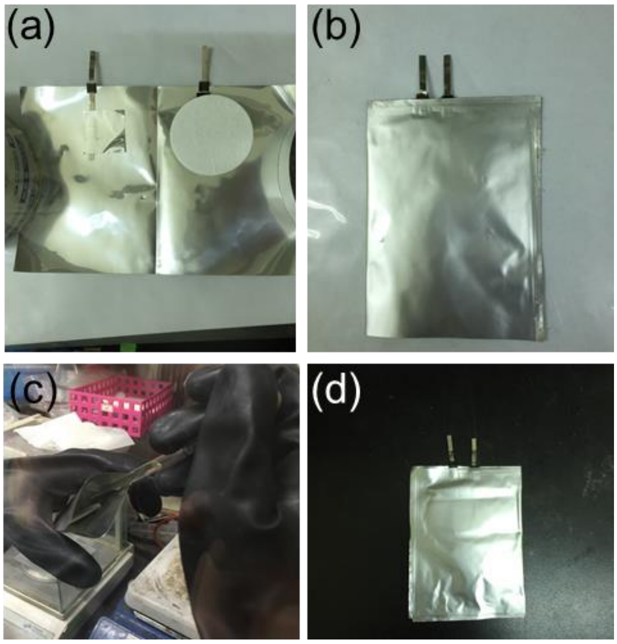

Supplementary Figure 6 | A detailed fabrication of Al/NG cell assembled in the pouch cell. (a) Pouch cells were assembled by using a NG cathode (4 mg cm^{-2}) and an Al foil (70 mg) anode, which were separated by one layer of glass fiber filter paper to prevent shorting. Polymer coated Ni bars were used as current collectors connected to NG film and Al foil by a carbon tape for the anode and cathode, separately. (b) The two sides of resulting pouch cell were sealed by a heating sealer for further adding electrolyte easily in the glove box. (c) The resulting pouch cell was delivered into the glove box. Then, the electrolyte (2mL, prepared using $AlCl_3$/[EMIm]Cl~1.3 by mole) was injected and the cell was sealed by a heating sealer. (d) Finally, the cell was prepared and removed from the glove box for further performance test.

Supplementary Table 1 | The comparisons of Intensity of [002] peak and D/G ratio of three commercial graphite and related battery performances. The data were extracted from Supplementary Figure 1.

Material	Intensity of [002] peak in XRD spectra	D/G ratio in Raman spectra	Discharge capacity (50th cycle)	Coulombic Efficiency
MCMB	4500	0.12	55	77%
KS6	1557	0.2	60	94%
SP-1 graphite	19400	0.041	110	96%

Supplementary Table 2 | Optimized geometries of $AlCl_3$ and $AlCl_4$ and carbon layer

System	Bare carbon	$AlCl_4$	$AlCl_4$	$AlCl_3$	$AlCl_3$	C-Cl
	d(C-C) (Å)	d(Al-Cl) (Å)	∠ (Cl-Al-Cl)	∠ (Cl-Al-Cl)	d(Al-Cl) (Å)	d(C-Cl) (Å)
Three layers	1.437~1.522					
Three $AlCl_4$		2.149<br>2.160<br>2.171<br>2.157	107.8°<br>106.8°<br>110.1°<br>107.6°	120.1°<br>121.0°<br>118.5°	2.165<br>2.158<br>2.159	1.711<br>1.687

Supplementary Methods

Theoretical calculations.

a. $AlCl_4^-$ anions at edge position of the carbon layer. All calculations in this work were performed using the CASTEP program,[1] which employs the plane wave pseudopotential method to calculate the total energy within the framework of the Kohn-Sham DFT.[2] Three flakes of graphene and five $AlCl_4^-$ anions were considered in the DFT calculation. The models were fully optimized, allowing the relaxation of all atoms. The system was gradually relaxed to achieve a balanced state with the lowest energy. In the charge/discharge process, $AlCl_4^-$ anions were supposed to move toward graphite layers. We used the RPBE exchange-correlation functional.[3] The ion-electron interaction was modeled by the non-local real space, ultrasoft pseudopotential[4] with a cutoff energy of 400 eV.

In the DFT calculations, we employed projector-augmented waves (PAW)[4-6] generalized gradient approximation (GGA)[7] as implemented in the Vienna ab initio simulation package (VASP).[8, 9] The clusters 5 $AlCl_4$ and carbon layers were modeled by a three-layer carbon lattice, with a supercell (dimensions of a = b = 2.5 nm, and c = 3.0 nm). All Al_xCl_y-carbon layer clusters

models were fully optimized, allowing the relaxation of all atoms. The system gradually become relaxed to achieve a balanced state with lowest energy. Total energy calculations were performed as a cluster calculation (k=0) and convergence was achieved when the residual total energy of 0.01 eV and the maximal force of 0.01 eV $Å^{-1}$ were reached. The optimized geometries of $AlCl_3$ and $AlCl_4$ and carbon layer are shown in Supplementary Table 2.
The bond lengths of Al-Cl are listed in Supplementary Table 2. $AlCl_4^-$ clusters with tetrahedral and planar quadrangle geometries were inserted into the relaxed graphite separately, as shown in Figure. 6. In the DFT section, the Al-Cl bond length lengths are listed in Supplementary Table 2. $AlCl_4^-$ clusters, with tetrahedron and planar quadrangle geometries, were separately inserted into the relaxed graphite, as shown in Figure. 6. The $AlCl_4^-$ cluster size, before intercalation into the graphene layer, was 4.88 Å. Besides, we modeled graphite as 3-layer graphene. The *d*-spacing of the graphite was measured to be 8.205 Å between the three graphene layers, (note: the *d*-spacing is 4.017 Å between two graphene layers), which is slightly larger than the d spacing of bulk graphite (3.4 Å). Our calculated result is based on d-spacing that allows for two graphene layers with Van der Waals radii. The simulated supercell consists of 201 carbon atoms and five $AlCl_4^-$ clusters which were used with k-Point, and model dimensions 20 × 20 × 25 $Å^3$. When compared to the $AlCl_4^-$ cluster in the vacuum space, the intercalated $AlCl_4^-$ cluster showed clear distortions in both the calculated Al-Cl bond lengths and bond angles. The distortion directly results from the Van der Waals interactions between the graphene layers, which leads to flattening of the $AlCl_4^-$ tetrahedron in response to pressure from the c-axis direction. The interlayer graphene spacing was substantially enlarged (6.23Å) after $AlCl_4^-$ intercalation, with the geometric size (4.79 Å) of the intercalated anion.

Supplementary References |

1. Milman, V. et al. Electronic structure, properties, and phase stability of inorganic crystals: A pseudopotential plane-wave study. *Int. J. Quantum Chem.* **77**, 895-910 (2000).
2. Fuchs, M. & Scheffler, M. Ab initio pseudopotentials for electronic structure calculations of poly-atomic systems using density-functional theory. *Comput. Phys. Commun.* **119**, 67-98 (1999).
3. Hammer, B., Hansen, L.B. & Nørskov, J.K. Improved adsorption energetics within density-functional theory using revised Perdew-Burke-Ernzerhof functionals. *Phys. Rev. B* **59**, 7413 (1999).
4. Vanderbilt, D. Soft self-consistent pseudopotentials in a generalized eigenvalue formalism. *Phys. Rev. B* **41**, 7892 (1990).
5. Blöchl, P.E. Projector augmented-wave method. *Phys. Rev. B* **50**, 17953 (1994).
6. Kresse, G. & Joubert, D. From ultrasoft pseudopotentials to the projector augmented-wave method. *Phys. Rev. B* **59**, 1758 (1999).
7. Perdew, J.P. et al. Atoms, molecules, solids, and surfaces: Applications of the generalized

gradient approximation for exchange and correlation. *Phys. Rev. B* **46**, 6671 (1992).

8. Kresse, G. & Hafner, J. Ab initio molecular dynamics for liquid metals. *Phys. Rev. B* **47**, 558 (1993).
9. Kresse, G. & Furthmüller, J. Efficient iterative schemes for ab initio total-energy calculations using a plane-wave basis set. *Phys. Rev. B* **54**, 11169 (1996).
10. Zhang, X., Sukpirom, N. & Lerner, M. M., Graphite intercalation of bis(trifluoromethanesulfonyl) imide and other anions with perfluoroalkanesulfonyl substituents. *Mater. Res. Bull.* **34**, 363–372 (1999).
11. Ozmen-Monkul, B. & Lerner, M. M., The first graphite intercalation compounds containing tris(pentafluoroethyl)trifluorophosphate. *Carbon* **48**, 3205–3210 (2010).
12. Nakaya, K et.al Oxidation of Nickel in AlCl3-1-Butylpyridinium Chloride at Ambient Temperature. *J. Electrochem. Soc.*, **162** (1) D42-D48 (2015).
